# A Review of the Pharmacological Effects of *Solanum muricatum* Fruit (Pepino Melon)

**DOI:** 10.3390/foods13172740

**Published:** 2024-08-29

**Authors:** Hei-Tung Lydia Chan, Ka-Man Chan, Abhreet-Kaur, Sze-Wing Sam, Shun-Wan Chan

**Affiliations:** Department of Food and Health Science, Technological and Higher Education Institute of Hong Kong, Hong Kong; lydiachan@thei.edu.hk (H.-T.L.C.); 210093391@stu.thei.edu.hk (K.-M.C.); 210291499@stu.thei.edu.hk (A.-K.); tasiasam@thei.edu.hk (S.-W.S.)

**Keywords:** *Solanum muricatum* fruit, antioxidant, anticancer, anti-inflammatory, antidiabetic, pharmacology

## Abstract

*Solanaceae*, commonly known as nightshade, is a diverse family of flowering plants comprising around 90 genera and an estimated 3000–4000 species. *Solanaceae* spp. and its various fruits, including pepino (*Solanum muricatum*), commonly known as pepino melon, are widely recognized by the public for their nutritional value and pharmacological effects. Pepino melon, in particular, is often enjoyed as a fresh dessert or salad due to its juicy flesh. Given its beneficial properties, the potential of pepino melon to be developed as a functional food has been extensively studied. This review aims to provide a comprehensive summary of the reported pharmacological effects of the active compounds found in pepino plant and melon. Among these compounds, polyphenols, notably quercetin, and vitamin C have demonstrated notable antioxidant properties such as scavenging free radicals, effectively protecting against free-radical damage. Moreover, these active ingredients provide pepino with anti-inflammatory properties by inhibiting the expression of proinflammatory cytokines and enzymes, thereby reducing nitric oxide production. Additionally, they have shown promise in selectively targeting cancer cells, exhibiting anti-cancer properties. Furthermore, the active compounds such as quercetin in pepino have been associated with anti-diabetic effects, improving insulin sensitivity and inhibiting insulin resistance. Overall, this review highlights the diverse and significant pharmacological effects of the active compounds found in pepino melon, emphasizing its potential as a valuable functional food.

## 1. Introduction

*Solanaceae*, commonly known as nightshade, is a diverse family of flowering plants comprising around 90 genera and an estimated 3000–4000 species. The plants in this family range from annual and perennial herbs to shrubs and small trees. They have played a significant role in human history, serving as a vital source of both food and medicine for thousands of years. Among the most widely cultivated and recognized nightshade foods are *Solanum tuberosum* (potato), *Solanum melongena* (eggplant), *Solanum lycopersicum* (tomato), and *Capsicum* spp. (pepper). The potato is a crop of significant economic importance, and it has emerged as one of the most vital food staples globally [1,2,3]. In addition to its importance in human food history, *Solanaceae* has also played a significant role in genetic research. The potato is a genetic basis model for studying tuber development, while the tomato serves as a genetic model for fruit development and composition. Pepper and eggplant are also important horticultural crops around the world, and genetic and genomic research in these plants has largely followed the tomato model [1].

**Figure 1:**
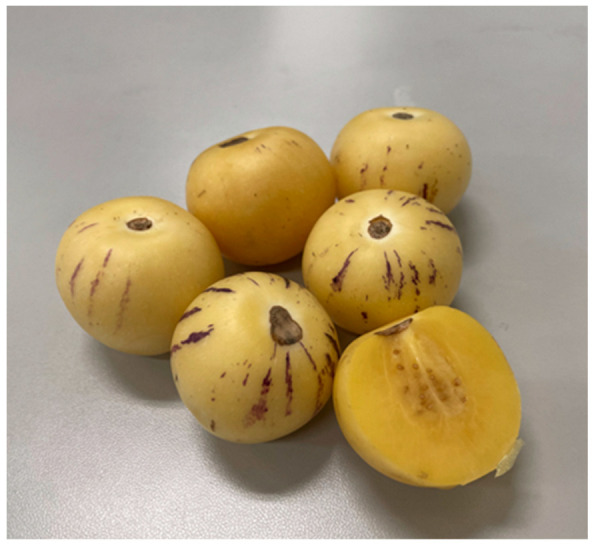
Photo of *Solanum muricatum* fruit (pepino melon).

One lesser-known member of the *Solanaceae* family is the pepino (*Solanum muricatum*), also known as sweet cucumber or melon pear, which is a member of the nightshade family [4]. Pepino, an Andean crop, possesses numerous names that can often be confused with cucumber. Etymologically, pepino has at least 16 distinct Spanish names, which can be traced back to the arrival of the Spaniards in South America. This historical development has contributed to the varied terminology associated with pepino [5,6]. Unlike other *Solanaceae* food plants such as potato, tomato, pepper, and eggplant, pepino melon is not widely known or widely cultivated. The cultivation of pepino, an annual crop in most regions and a pluriannual crop in frost-free areas, is often overlooked due to its susceptibility to environmental conditions, particularly temperature. The fruit set and quality of pepino are highly sensitive to these factors. Additionally, the ripening process of pepino melon can be time-consuming. Consequently, numerous studies conducted across various countries, including Spain, Italy, China, and others, have focused on enhancing the cultivation of pepino. These studies aim to increase pepino yields and improve its overall quality. Modern tomato growing techniques are being introduced for the cultivation of pepino, as both crops belong to the same family. By implementing these techniques, farmers can leverage the knowledge and practices developed for tomato cultivation to enhance the growth and productivity of pepino [5,6]. Furthermore, a study is being conducted to provide support to farmers in different countries by supplying them with improved varieties of pepino. The objective is to enhance the commercial production of pepino fruit and promote its growth on a larger scale [7]. Pepino is valued as a medical plant, with studies showing that the aqueous extract of its leaves has antioxidant, anti-obesity, anti-diabetic, and anti-inflammatory effects. Scientific research has demonstrated that this extract can reduce endoplasmic reticulum stress, protect against palmitic acid-induced lipotoxicity, inhibit apoptosis, and improve insulin sensitivity [5,6,8,9]. In addition to its leaves, the pepino fruit also holds a high nutritional value. Its fruit has a distinctive appearance, with golden yellow skin that may or may not have purple stripes, and a round, elongated, or ellipsoid shape (Figure 1) [7,8,9,10,11]. The fruit has a juicy yellow flesh with an attractive flavour and aroma, making it a refreshing dessert fruit because of the melon-like flavour when pepino fruit is ripe, and as a salad ingredient similar to cucumber when pepino fruit is less ripe [7,11].

This review article summarizes the latest findings on the pharmacological effects of pepino, with the aim of attracting the interest of researchers towards to this potential functional food.

## 2. Methodology

The search for relevant literature was carried out electronically on the specified scientific databases: (1) Science Direct; (2) MDPI; (3) ProQuest; (4) National Institutes of Health; and (5) PubMed (provided on behalf of the Technological and Higher Education Institute of Hong Kong). The searches were conducted across each database using 1 Boolean Code:

(Solanum muricatum* Pharmacological effect*) or (Solanum muricatum* nutrition*) or (Solanum muricatum* composition) or (pepino* Pharmacological effect*) or (pepino* nutrition*) or (pepino* composition).

The search was restricted to peer-reviewed and published journal articles exclusively, with no inclusion of grey literature. There were no constraints on publication date, geographical origin, or field of study; however, articles had to be in English. The reviewers have screened 783 articles, of which 26 were considered suitable for including in the current review article.

## 3. Nutritional Value

In recent years, there has been growing interest in pepino melon due to its nutritional value, as it is rich in bioactive compounds such as vitamins, polyphenols and antioxidants. Research has shown that the fruit contains a variety of antioxidant components that can provide health benefits to humans [12,13]. Studies have demonstrated that the pepino melon contains a high number of phenolic compounds, which may offer potential health benefits including antioxidative, anti-inflammatory, anti-cancer, and anti-diabetic effects [14,15].

Pepino melon is a fruit that is low in calories and sugar, due to its high water content (around 92%). Its main carbohydrates are fructose, glucose, and sucrose, with fructose being the most abundant when the fruit is still green. As the pepino melon ripens, the sucrose content increases, while fructose and glucose decrease. Research has also identified a novel *Solanum muricatum* polysaccharide (SMP-3a) that has been shown to promote macrophage proliferation of RAW264.7 cells and stimulate the secretion of tumour necrosis factor-alpha (TNF-α), interleukin-6 (IL-6), and IL-1β with an increasing concentration of SMP-3a [16,17,18,19,20]. Additionally, pepino melon contains anthocyanins and carotenoids, including β-carotene and xanthophyll. A study examining 15 different varieties of pepino melons found that the amount of β-carotene ranged from 56 to 166 mg per 100 g of fruit [18,21,22,23,24]. Regular pepino melon consumption could significantly contribute to the recommended daily allowance (RDA) of phosphorus, potassium, iron and copper. The study found that pepino melon is rich in these essential minerals, with a 200 g serving containing varying amounts of potassium (ranging from 99.8 to 353.8 mg), phosphorus (ranging from 18.0 to 51.1 mg), calcium (ranging from 5.5 to 17.9 mg), magnesium (ranging from 5.7 to 9.4 mg), iron (ranging from 0.2 to 0.6 mg), copper (ranging from 0.01 to 0.09 mg), and zinc (ranging from 0.1 to 0.2 mg) [22,23,25]. In addition, pepino melon is also rich in polyphenols, including phenolic acid and flavonoids, which have been linked to potential health benefits such as antioxidant, antidiabetic and anti-inflammatory effects. Some studies suggest that the phenolic content of ripened pepino melon is higher than that of the unripened one [11,19,20,23,24,26,27,28,29]. Moreover, pepino also contains essential vitamins such as thiamin, niacin, and riboflavin, as well as vitamin C and organic acids such as citric acid and malic acid, which provide numerous metabolic and antioxidant benefits [23,26]. A study has shown that pepino melon contains a significant amount of vitamin C, ranging from 30 to 80 mg per 100 g, which is higher than some tomato varieties, despite both plants belonging to the same family, *Solanaceae* [11,18,19,20]. Additionally, a study has shown that pepino melon contains a significant amount of phosphatidylcholine (18:2), which is used for non-alcoholic fatty liver disease treatment [20]. It has been demonstrated that that 27 key flavour-related metabolites play a crucial role in distinguishing the origins of pepino melon [30], with variations in soil nutrients contributing to the metabolite variability in pepino melon [19]. As a result, the quantity and quality of active ingredients present in pepino melon heavily depend on the geographical source of its cultivation regions.

In our study, pepino melons were purchased from three major local markets in Hong Kong. The majority of these pepino melons were round, with a slightly flattened shape, ranging from 43 to 83 mm in diameter and weighing between 135.6 and 300.0 g. The juice yield of these melons varied from 70.0 to 82.0%, with dry matter content ranging from 2.18 to 4.79 g. The Brix (total soluble solids, SSC) content fell between 6.1 and 11, and pH values ranged from 5.5 to 6.0. It is worth noting that a study indicated that pepino melons could be egg-shaped, weighing between 210 and 370 g, with diameters of 60 to 125 mm, and a juice yield varying from 60 to 68% [31].

## 4. Active Ingredients Found in *Solanum muricatum* Fruit

Polyphenols, especially quercetin, and ascorbic acid are the major active ingredients separated from pepino melon. There are 30 different constituents of polyphenol identified from pepino melon (Table 1). The reported pepino melon’s pharmacological effects contributed by individual active ingredients are also summarised in Table 1. Table 2 lists the major nutrients found in pepino melon. Comparative analysis of plant parts, extract types, study types and pharmacological effects of *Solanum muricatum* reported in the literature are summarised in Table 3.

## 5. Pharmacological Effects

**Figure 2:**
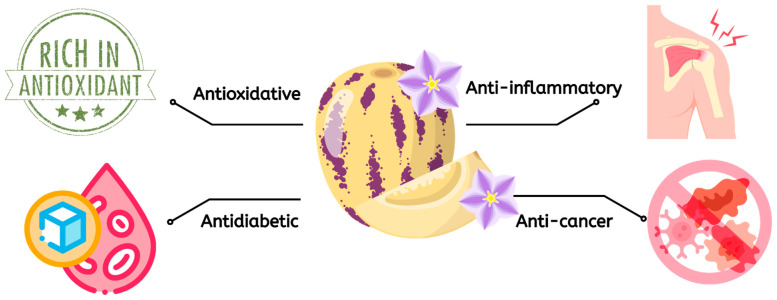
Health benefits of the fruit of *Solanum muricatum*.

*Solanum muricatum* has been reported for its biological activities and pharmacological effects including antioxidant, anticancer, antidiabetic and anti-inflammatory activity (Figure 2).

### 5.1. Antioxidant Activity

The antioxidative characteristics of both the aqueous extract of pepino melons and leaves has been examined in several recent experimental studies. Hydroxycinnamic acid (HCA) seems to be one of the most significant polyphenols that exhibit antioxidant properties by scavenging intracellular reactive oxygen species (ROS) [23]. Apart from phenolic acid, a considerable amount of flavonoid has also been detected in pepino melon extract through the inhibition of oxidative stress, especially lipid peroxidation. Moreover, Sudha et al. have discovered that the degree of maturity may influence the total phenolic content and antioxidant activity of pepino, in studies in which ripened fruit displayed a greater antioxidant effect than raw fruit in general [26,29].

Because of its antioxidant properties, research has suggested using aqueous extract of pepino leaves to treat alcoholic hepatic injuries by inhibiting lipid peroxidation in liver [6]. It has been hypothesized that the presence of polyphenols may benefit in suppressing the expression of lipogenic enzymes targeted on the liver to regulate lipid homeostasis. Meanwhile, the diversified phenolic profile of pepino fruits can be advantageous for lowering the risk of developing other oxidative stress-related diseases such as cardiovascular and neurological diseases, cancer, etc.

Other than fresh fruits, food processing techniques may lead to changes in nutritional composition and antioxidant properties of pepino melons. According to Scala et al. (2011), the significant loss of ascorbic acid and phenolic compounds in dehydrated fruit samples is due to the use of convective drying [31]. In the contrary, microwave-dried pepino fruits tend to preserve more antioxidant metabolites compared to other drying methods [39]. However, there is still a lack of investigations explaining the mechanism behind this at the present moment.

In addition, the flesh of the pepino melon contains other antioxidant components like carotenoids and vitamin C, which may support the antioxidant activity [23]; carotenoids and vitamin C may also protect against free-radical-mediated damage, lowering the probability of chronic diseases [40]. Moreover, vitamins and minerals can be found in pepino melon, including thiamine, niacin, and riboflavin, which also have high antioxidant activity [19]. Further, antioxidant and radical-scavenging activity are demonstrated by the ethyl acetate extract from raw pepino [29]. According to Sudha et al. (2011), the ethyl acetate extract from pepino comprises a significant amount of phenolics and flavonoids, and hence ethyl acetate extract can be utilized as a natural antioxidant source [29].

### 5.2. Anti-Cancer Effects

Research conducted over the years suggests that pepino may exhibit anti-cancer activities. According to Ren and Tang (1999), via apoptosis induction, tumour’ growth in vivo and in vitro can be halted by extracts from pepino. Through the in vivo studies, it has been suggested that injecting pepino directly into the tumour mass can significantly decrease the tumour volume in nude mice inoculated with MKN45 gastric cancer cells, which was one of the tumour cell lines being tested; pepino possesses selective cytotoxic activity [41].

Moreover, pepino melon-extract treatment can noticeably inhibit the formation of lung tumour nodules; it can also decrease the lung hydroxyproline, hexosamine, and uronic acid levels (*p* < 0.01) implying tumour growth reduction [15]. Shathish et al. (2015) have analysed that, compared to the untreated control group, the metastatic tumour-bearing animals treated with pepino melon extract concurrently had lung nodule inhibition of 73.6% [15].

Furthermore, after pepino melon extract administration, it has been observed that the life span percentage has increased; meanwhile, the growth of solid tumours has been substantially inhibited. A study stated that pepino melon-extract treatment could decrease glutathione (GSH) levels, which is conjectured to be used by cancer cells to safeguard against oxidative damage, in Dalton’s Lymphoma Ascites (DLA) inoculated mice [35]. Moreover, pepino melon extract can also significantly lower the significantly elevated nitric oxide (NO) and TNF-α levels. The TNF-α level in the tumour control group (802.6 ± 12.0) drastically (*p* < 0.01) declined, to 175.2 ± 16.5, during the treatment with pepino melon extract [35]. Nitric oxide may cause vasodilation, increasing blood flow to the tumour. TNF-α was involved in the up-regulation of nitric oxide synthesis, and the primary source of TNF-α includes macrophages, T-lymphocytes, natural killer (NK) cells, and proliferating B cells. Hence, pepino melon extract can be considered an anti-cancer agent [35].

### 5.3. Antidiabetic Activity

Several research studies have proposed that flavonoid and other phenolic compounds in pepino melon extract can possibly ameliorate high blood glucose level and insulin resistance. Quercetin is a type of dietary flavonoid with the potential effect of improving insulin sensitivity and glycaemic control. According to Hussain et al. (2019), pepino herb extract contains 0.87 ± 0.05% quercetin. This flavanol is considered to be an efficacious agent for activating AMP-activated protein kinase to inhibit insulin resistance [9]. Moreover, quercetin and some other polyphenols are likely to contribute to glucose homeostasis, with an increased expression of glucokinase (GLK) [42]. GLK protein is responsible for regulating glucose metabolism. In addition, pectic substances can help to diminish both fasting blood glucose and postprandial blood glucose [42,43]. Mature pepino fruit contains approximately 9.60–10.10 mg of water-soluble pectin per gram of dry weight. It is possible that these water-soluble dietary fibres help reduce digestion rate, resulting in a lower glycaemic (GI) index. Furthermore, pepino melon extract may benefit the improvement of insulin sensitivity by inhibiting the resistin expression of adipocytes [27]. A number of studies have examined the association between resistin expression, diabetes and obesity previously, while Jamaluddin et al. (2012) have further added that a high serum resistin level increases cardiovascular-related mortality among diabetes patients [44].

On the other hand, the antioxidative properties of some flavonoid and polyphenolic compounds in pepino melon extract also possibly attenuate diabetic deterioration and diabetes-associated complications. These metabolites can be viewed as ‘free radical scavengers’, which inhibit vascular oxidative injury by eliminating ROS [45]. The effect of pepino fruits on restoring GSH and glutathione peroxidase (GPX) synthesis may further promote glucose homeostasis and ameliorate accumulated cardiac oxidative damage and microvascular complications [27,46]. Moreover, this medicinal plant can potentially downregulate diacylglycerol acyltransferase1 (DGAT1) expression to mediate excessive triglyceride (TC) synthesis [27] and lowered cholesterol concentration [47]. Another example of preventing diabetic complications would be diabetic neuropathy. Research has shown that increasing the level of GSH and GPX seems to offer significant protective factors to preserve the peripheral nerve in the human body [48]. The neuroprotective effect may be due to the amount of coumaric and caffeic-acid derivatives. Serina and Castilho (2021) have further illustrated the point that polyphenolic compounds may protect nerve fibres from demyelination induced by neuroinflammation and accumulated oxidative stress [49].

While looking through the lens of disease prevention, pepino melon is suitable for people with diabetes because of its low GI index. Despite the fact that the total sugar content of pepino fruits increases, with sucrose as the predominant form of free sugar, as ripening progresses [19,50], moisture remains as the major component in pepino melons, contributing over 90%, approximately, of fruit composition [50]. As the total solid carbohydrate concentration reduces, this may be beneficial for postprandial glycaemic control. Meanwhile, the presence of dietary fibre (pectin) is worth noticing.

### 5.4. Anti-Inflammatory Effects

Inflammation is a defensive reaction of the immune system that eliminates harmful or toxic stimuli and initiates the healing process. As such, it is a vital defence mechanism that helps maintain overall health. Inflammation can be triggered by non-infectious factors like physical injury and chemical irritants, as well as infectious factors. It can be categorized into acute inflammation and chronic inflammation, which can arise due to uncontrolled acute inflammation or as a result of autoimmune disorders or diseases, and which may persist for weeks, or even years [35,51]

Pepino has been reported to have an anti-inflammatory effect in several studies, and blockage of the inflammation-mediator release is one of the crucial mechanisms of pepino’s anti-inflammatory action. For instance, one study demonstrated significantly reduced paw oedema in rats, induced by carrageenan and formaldehyde, suggesting that it may be a potent inhibitor of acute and chronic inflammation by blocking the release of inflammation mediators such as prostaglandin or bradykinin [35]. In the same study, repression of proinflammatory cytokine expression also proved to be one of the essential mechanisms of pepino’s anti-inflammatory action. The result proves that the increased TNF-α was significantly decreased after the pepino treatment, which is consistent with other studies indicating that pepino can inhibit various cytokines such as TNF-α, IL-6, IL-1β, IL-2, and granulocyte monocyte colony-stimulating factor (GM-CSF); this may be due to the presence of quercetin, which has been shown to inhibit COX-2 and IL-6 expression and increase ovarian content of TNF- α in a rat model [14,15,35,48,51,52]. Moreover, pepino melon extract has been reported to inhibit the activation of the subunit (p65 and p50) of nuclear factor-kappaB (NF-κB), which plays a key role in the pro-inflammatory gene expression, including cytokines, as well as inhibiting its nuclear translocation. Also, a study shows that pepino contains quercetin, which is proven to significantly inhibit the expression of NFκb [14,15,52,53].

Inflammatory enzyme expression, including inducible nitric oxide synthase (iNOS) and cyclooxygenase (COX)-2, plays a critical role in the development of inflammatory-related diseases such as cancer and cardiovascular disease. In response to immune activation, iNOS is induced by various agents such as lipopolysaccharide and cytokines, leading to the generation of high concentrations of NO, over time. These high levels of NO can act as a toxic- or immune-regulatory mediator, suppressing activation and causing the death of inflammatory cells. However, at low concentrations, NO can be pro-inflammatory [54]. On the other hand, the increase in NO production stimulates the COX-2 expression causing the formation of prostaglandin E2, which is a principal inflammatory mediator [55,56]. Hence, inhibition of inflammatory enzyme expression and production of NO are potential treatments for inflammatory disease. There is scientific evidence showing that pepino melon extract significantly inhibits the production of NO, which is lipopolysaccharide-stimulated, in macrophages, and also decreases the serum NO level and the expression of iNOS and COX-2 in mice lung tissue by ~30% and 40%, respectively. A study also shows that pepino contains chlorogenic acid, which is known to inhibit iNOS [15,23,37]. Furthermore, a study demonstrates that a novel polysaccharide SMP-0b from pepino melon extract shows a strong immunomodulatory effect [32]. On the other hand, a study indicates that pepino contains phosphatidylcholine, which is used for non-alcoholic fatty liver disease, which can reduce inflammation [24]. Therefore, there has been evidence suggesting that pepino has potential as a functional food with significant anti-inflammatory activity; however, more detailed studies are needed to clarify the bioactive substance that is the potent anti-inflammatory agent in pepino.

### 5.5. Other Activities

Pepino herb extract has been identified as a potential new treatment for bone formation and remodelling. Studies have shown that treatment of rat bone marrow stromal cells (BMSCs) with pepino herb extract promotes the wingless-type MMTV integration site family (Wnt) and bone morphogenetic protein (BMP) signalling pathways, which are important for osteogenesis and which promote osteogenic differentiation in BMSCs [37,38]. Furthermore, another study demonstrated the effect of pepino herb extract on an osteogenesis imperfecta mouse model, which resulted in a significant increase in collagen content due to the promotion of collagen biosynthesis and inhibition of collagen degradation [36].

## 6. Perspectives

The saying “prevention is better than cure” holds particular significance in the era of an aging population. Therefore, it is crucial to explore the potential of *Solanum muricatum* fruit (pepino melon) in disease prevention, so as to slow down the aging process. While the nutritional value of this fruit has been established, further research is necessary to gain a comprehensive understanding of the specific active ingredients, such as polyphenols, responsible for their antioxidant, anti-inflammatory, antidiabetic, and anticancer functions. By conducting more detailed mechanistic studies, we can identify the sophisticated relationship between different polyphenols present in pepino melon and how they relate to their pharmacological effects. It is also interesting to evaluate the fruit’s potential in some unique areas, such as its antiglycation effect and impact on bone formation and remodelling.

Despite the abundance of polyphenols in pepino melon highlighted in numerous studies, there remains a significant lack of chemical analysis evidence to distinguish all its polyphenols. Furthermore, limited scientific research on the potential toxic effects or safety profile of pepino melon in various applications underscores gaps in understanding the associated risks and safety considerations. Conducting a thorough chemical analysis to identify and quantify the diverse polyphenols in pepino melon can offer crucial insights into its potential health-promoting properties, guiding further research, product development, and dietary recommendations. Performing systematic toxicity evaluation can provide essential data on its safety profile, to benefit the development of pepino melon as a functional food.

Exploring the synergistic effects of these polyphenols within pepino melon is vital to comprehend how they interact and collectively enhance health benefits, potentially surpassing the effects of individual compounds. Investigation into these synergies could shape the development of functional foods, dietary supplements, or pharmaceutical products that leverage the combined advantages of these compounds, advancing our understanding of pepino melon’s health-promoting potential and its applications in nutrition and therapy. Machine learning, combined with metabolomics and sensory evaluation, has been employed to identify key flavour-related metabolites in pepino melon [30]. This approach can be applied to identify new active ingredients contributing to any potential pharmacological effects.

The aging problem is a global issue that we should be aware of, especially how inflammatory conditions can contribute to age-related diseases. In this review, there are studies stating that pepino melon is an excellent source for obtaining antioxidant and anti-inflammatory agents, as a potential functional food, with scientific support mainly from in vivo and in vitro experiments. Because of the lack of human study, it is highly recommended that researchers launch human studies with an emphasis on the inflammatory cytokines (IL-6, IL-1β, TNF-α) in blood, to check the anti-inflammatory effect. This can provide substantial evidence on the health benefits of pepino melon for human health, aiding in formulating recommendations for its utilization in dietary or therapeutic contexts. Further investigation on the effects of pepino melon on gut microbiome may be another good direction to take to explain the therapeutic effects, such as the anti-inflammatory effect of pepino melon, as a similar relationship has been reported in other fruits, such as blueberries [57], strawberries [58], bitter melon [59], and grapes [60].

By exploring these aspects more deeply, we can acquire valuable insights into the preventive properties and broader health benefits of pepino melon, thereby addressing the needs of an ageing population more effectively.

## Figures and Tables

**Table 1:** Polyphenols found in the fruit of *Solanum muricatum* and their reported pharmacological effects related to the fruit of *Solanum muricatum*.

Classification	Compound	Molecular Formula	MW	Effect	Reference
Flavonoids	Quercetin	C_15_H_10_O_7_	302.23	Improving insulin sensitivity; inhabitation of COX-2, IL-6 and NFκb; antioxidant	[8,14,27]
	Quercetin-3-O-galactoside; Hyperoside	C_21_H_20_O_12_	464.4		[28]
	Myricetin	C_15_H_10_O_8_	318.23		[14]
	Naringenin	C_15_H_12_O_5_	272.25		[14]
	Rutin	C_27_H_30_O_16_	610.5		[14]
Chlorogenic acids	1-Caffeoylquinic acid 3-Caffeoylquinic acid 4-Caffeoylquinic acid 5-Caffeoylquinic acid 2-Caffeoylquinic acid	C_16_H_18_O_9_	354.3087	Anti-inflammatory	[23,28]
	di-Caffeoylquinic acid	C_25_H_24_O_12_	516.45		[23]
	3-O-p-Coumaroylquinic acid 5-O-p-Coumaroylquinic acid	C_16_H_18_O_8_	338.312		[28]
Hydroxycinnamic acids	Caffeic acid	C_9_H_8_O_4_	180.16	Antioxidant	[28]
	1-O-caffeoyl-beta-D-glucose	C_15_H_18_O_9_	342.30		[28]
	4-caffeoylshikimic acid 3-caffeoylshikimic acid	C_16_H_16_O_8_	336.2934		[28]
	1-O-Feruloylglucose	C_16_H_20_O_9_	356.32		[23]
	Glucosyringic acid	C_15_H_20_O_10_	360.31		[28]
Hydroxycinnamic acids derivatives	Rosmarinic acid	C_18_H_16_O_8_	360.3		[28]
Vanillic acids	Vanillic acid-4-O-glucuronide	C_14_H_16_O_10_	344.27		[28]
Ferulic acids	6-O-Feruloyl-beta-D-glucose	C_16_H_20_O_9_	356.32		[28]
Coumarins	Skimmin	C_15_H_16_O_8_	324.28		[28]
	Umbelliferone; 7-Hydroxycoumarin	C_9_H_6_O_3_	162.14		[28]
	7-methoxycoumarin	C_10_H_8_O_3_	176.17		[28]
Lignans	Vitexdoin E	C_19_H_16_O_6_	340.3		[28]
Phenolic compounds	Mucic acid dimethyl ester	C_8_H_14_O_8_	238.19		[28]
	Arbutin	C_12_H_16_O_7_	272.25		[28]

**Table 2:** Major nutrients found in the fruit of *Solanum muricatum*.

Types	Nutrient Ingredients	References
Vitamins	B_1_, B_2_, B_3_, C	[10,18,20,24]
Carbohydrates	polysaccharide SMP-3a, SMP-0b, dietary fibre including pectin, monosaccharides including sucrose, fructose, glucose	[16,18,20,32,33,34]
Amino acids	60 amino acid and their derivatives, including 7 essential amino acids (tryptophan, phenylalanine, methionine, threonine, isoleucine, leucine, and valine)	[19,24,34]
Lipids	36 different type of fatty acid, 16 lysophosphatidylcholine (LPC), 6 lysophosphatidylethanolamine (LPE), phosphatidylcholine (18:2) (PC)	[19,24]
Trace minerals	Potassium, Phosphorus, Iron, Copper, Calcium, Zinc	[20,23]
Organic acid	Citric acid, Malic acid	[16,18,34]
Carotenoids	α-carotene, β-carotene, xanthophyll (zeaxanthin and lutein), β-apo-8-carotenal, α-cryptoxanthin, β-cryptoxanthin, lycopene	[17,21,22,23]
Others	Plumerane, Anthocyanins	[24]

**Table 3:** Comparative analysis of plant parts, extract types, study types and pharmacological effects of *Solanum muricatum* reported in the literature.

Part of the Plant	Type of Extract	Type of Study	Effect
Leaf	Aqueous extract	In vivo	Antioxidant activity [6], Antidiabetic activity [8]
		In vitro	Antioxidant activity [5]
	Methanol extract	In vitro	Antidiabetic activity [9]
Pulp	Aqueous extract	In vitro	Anti-inflammatory [20]
	Acidic extract	In vitro	Antioxidant activity [23], Anti-inflammatory [23]
	Ethanol extract	In vitro	Anti-inflammatory [20]
Fruit	Aqueous extract	In vivo	Antidiabetic activity [27], Anti-inflammatory [14]
	Ethanol extract	In vivo	Anti-inflammatory [14]
		In vitro	Antioxidant activity [26], Anti-inflammatory [32]
	Ethyl acetate extract	In vitro	Antioxidant activity [29]
	Methanol extract	In vivo	Anti-cancer [15,35], Anti-inflammatory [15,35]
Whole plant	Aqueous extract	In vivo	Osteogenesis activity [36]
		In vitro	Osteogenesis activity [37,38]

## Data Availability

No new data were created or analyzed in this study. Data sharing is not applicable to this article.
